# Tides and Their Dynamics over the Sunda Shelf of the Southern South China Sea

**DOI:** 10.1371/journal.pone.0162170

**Published:** 2016-09-13

**Authors:** Farshid Daryabor, See Hai Ooi, Azizan Abu Samah, Abolghasem Akbari

**Affiliations:** 1 National Antarctic Research Center, Institute of Postgraduate Studies, University of Malaya, 50603, Kuala Lumpur, Malaysia; 2 Institute of Ocean and Earth Sciences, Institute of Postgraduate Studies, University of Malaya, 50603 Kuala Lumpur, Malaysia; 3 Faculty of Civil Engineering and Earth Resources, University Malaysia Pahang, Lebuhraya Tun Razak, 26300 Gambang, Kuantan, Pahang, Malaysia; Centro de Investigacion Cientifica y de Educacion Superior de Ensenada Division de Fisica Aplicada, MEXICO

## Abstract

A three-dimensional Regional Ocean Modelling System is used to study the tidal characteristics and their dynamics in the Sunda Shelf of the southern South China Sea. In this model, the outer domain is set with a 25 km resolution and the inner one, with a 9 km resolution. Calculations are performed on the inner domain. The model is forced at the sea surface by climatological monthly mean wind stress, freshwater (evaporation minus precipitation), and heat fluxes. Momentum and tracers (such as temperature and salinity) are prescribed in addition to the tidal heights and currents extracted from the Oregon State University TOPEX/Poseidon Global Inverse Solution (TPXO7.2) at the open boundaries. The results are validated against observed tidal amplitudes and phases at 19 locations. Results show that the mean average power energy spectrum (in unit m^2^/s/cph) for diurnal tides at the southern end of the East Coast of Peninsular Malaysia is approximately 43% greater than that in the East Malaysia region located in northern Borneo. In contrast, for the region of northern Borneo the semidiurnal power energy spectrum is approximately 25% greater than that in the East Coast of Peninsular Malaysia. This implies that diurnal tides are dominant along the East Coast of Peninsular Malaysia while both diurnal and semidiurnal tides dominate almost equally in coastal East Malaysia. Furthermore, the diurnal tidal energy flux is found to be 60% greater than that of the semidiurnal tides in the southern South China Sea. Based on these model analyses, the significant tidal mixing frontal areas are located primarily off Sarawak coast as indicated by high chlorophyll-*a* concentrations in the area.

## Introduction

**Fig 1:**
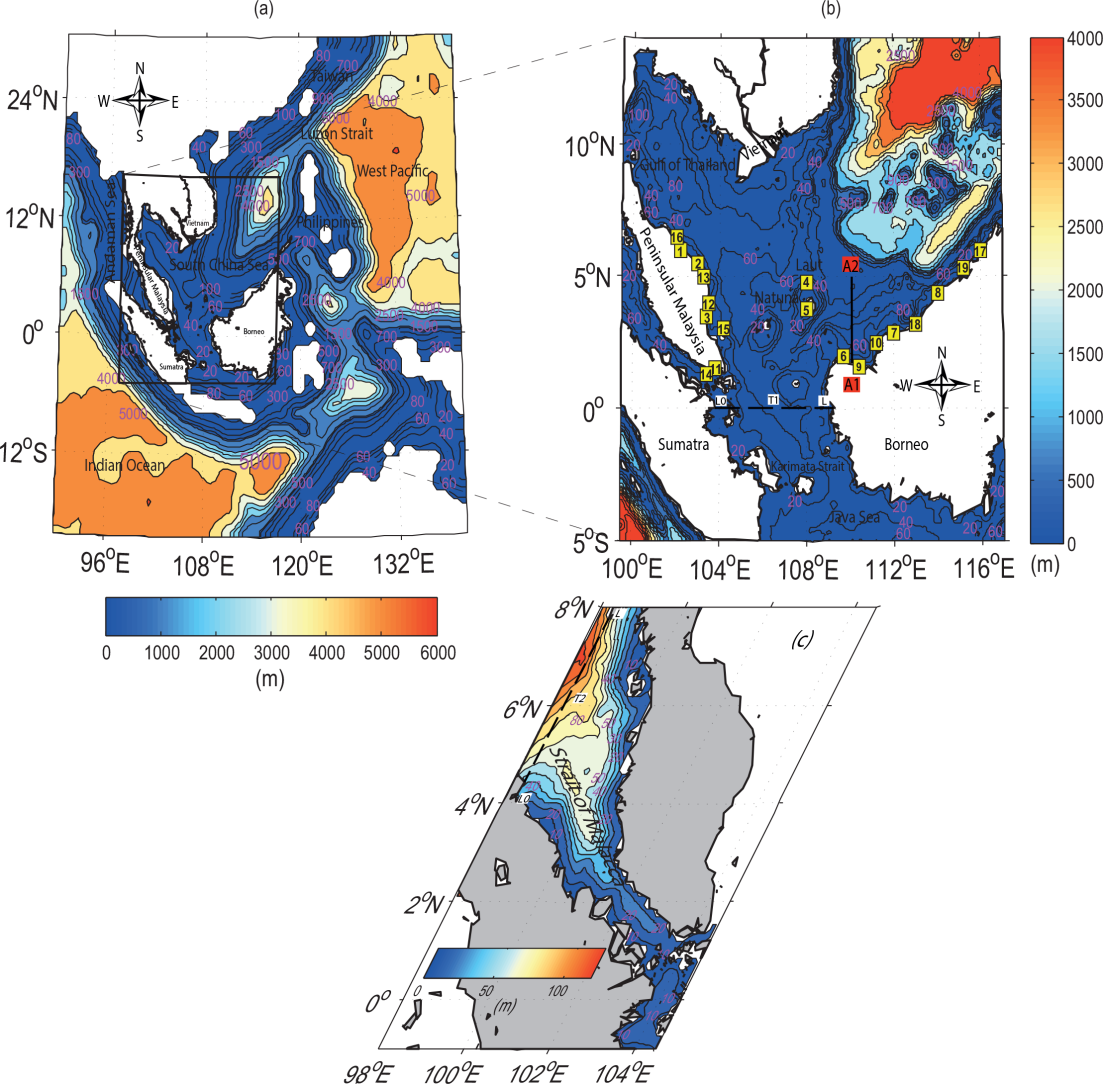
Bathymetry in (*a*) the coarse resolution and (*b*) the fine resolution domains of the two-domain nested model. The boundary of the nested domain is presented as a black box in (*a*). The numbers in yellow squares indicate the tidal stations (see Table 2 for station identifications), where the tidal harmonic constants are available for model verification. The dashed line marked with T1 at (*b*) indicates the pathway between the mouth of SSCS and the Java Sea, transect of A1A2 is used to evaluate the bi-monthly (January-February) variations of density and nutrients (such as phosphate) in the water column. (*c*) Bathymetry of the Strait of Malacca is marked by the dashed line T2 at the head of the Strait to estimate the resonance frequency.

The South China Sea (SCS) is a semi-enclosed tropical sea, located between several land-masses that include Peninsular Malaysia, Borneo, the Philippines and East Asia. The SCS has a complex bathymetry with a depth ranging from over 1000 m in the middle and northern parts to less than 100 m in the continental shelf (Fig 1). The southern South China Sea (SSCS) is bounded by Peninsular Malaysia’s eastern continental shelf, the Gulf of Thailand and the sea off Borneo and the southern coast of Vietnam. It is connected to the Java Sea through the Sunda Shelf in the south. Monsoonal winds have great influence on the sea circulation in the SSCS [1–11]. Furthermore, sea surface and seabed also have different significant impacts on wind-induced circulations as well as on the distribution, propagation and dissipation of tidal energy flux [12] with complicated tidal dynamics, especially at the bottom [13–15]. In this region, several successful 2D numerical studies have been performed by Ye and Robinson [16] on M_2_ and K_1_ tidal constituents with approximately 34 km resolution, Fang *et al*. [17] on M_2_, S_2_, K_1_, and O_1_ with 28 km resolution. Zu *et al*. [18] used simulation model of 10 km resolution and ETOPO5 with a model integration of 240 days to investigate characteristics and dynamics of M_2_, S_2_, K_1_, O_1_, N_2_, K_2_, P_1,_ and Q_1_. Most recently, Green *et al*. [19] used the Oregon State University Tidal Inversion Software (OTIS) without data assimilation but with a realistic tidal conversion scheme to demonstrate that the modelled dissipation levels are overestimated over the entire SCS, noting that the discrepancies are far larger in regions with steep bathymetry.

All these studies illustrate that the existing tidal currents within the continental shelf are strong and complex. In contrast to the northern region of the South China Sea, the dynamics of the SSCS have yet to be thoroughly investigated. The lack of a detailed modelling study and sufficient observations in the SSCS region justifies the need for such a study. Hence, the aims of this study are to understand and simulate tidal characteristics and their dynamics in the region using the Regional Ocean Modelling System (ROMS), as well as to validate the model against the observed values from tide gauges (TGs). After describing the model set up, we discuss the model validation and the basic tidal features, inclusive of its dynamics and mixing fronts. The final section summarizes our study.

## Model Configuration and Validation

### Model configuration

A two-domain, one-way nested model is configured for the SSCS (Fig 1). The model is based on ROMS (refer to: https://www.myroms.org/wiki/index.php/Documentation_Portal), which is commonly used for coastal applications [20], and the developed version (ROMS AGRIF) by the Institut De Recherche Pour Le Développement (refer to: http://www.romsagrif.org) is utilized for simulation here. The bathymetry of the outer and inner domains is based on ETOPO2 (refer to: http://www.ngdc.noaa.gov), which is derived from the depth soundings and satellite gravity observations [21]. However, the bathymetry is smoothed to reduce the pressure gradient error to an acceptable level by using the relative bathymetric gradient (*r* = *∇h/h*) to be 0.2 [6–7, 11]. The outer domain has a horizontal resolution of 0.25°×0.25° which is approximately 25 km and is vertically separated into 30 *S*-levels following the bathymetry but with a minimum depth (hmin) setting of 5 m at the shore. The outer domain covers 20° S to 30° N and 90° E to 140° E, thus encompassing the eastern Indian Ocean and the western Pacific Ocean. The inner domain covers 5° S to 14° N and 99.5° E to 117° E, thus encompassing the SSCS (Fig 1B). It shares the same vertical levels as the outer domain but utilizes a finer horizontal grid which is 0.083°×0.083°, each representing approximately 9 km. Overall, the dimensions are 199×207×30 and 239×207×30 for the outer and inner domains respectively.

The *K*-profile parameterization scheme, which includes important physics of the upper ocean mixing, is used for the vertical mixing processes [22]. The lateral boundary conditions for the inner domain are provided by integrating the outer domain according to the time step [23], thus making it into a one-way nested model. The leapfrog integration of outer and inner domains are based on the Adaptive Grid Refinement in FORTRAN (AGRIF) [24], as provided by the Institute De Recherche Pour Le Développement (refer to: http://www.romsagrif.org). It uses boundary conditions from the coarse outer domain for the fine resolution in the inner domain. The four open boundaries are specified at the north and south, east and west of both domains respectively (see Fig 1A). The simulation included both barotropic and baroclinic modes. The barotropic mode calculates the 2D momentum and elevation fields, whereas the baroclinic mode computes the 3D velocity and tracer (such as temperature and salinity) fields. Flather’s [25] and Chapman’s [26] boundary conditions are used for the 2D momentum and elevation fields respectively. The Orlanski [27] radiative boundary condition is used for the 3D fields.

The tidal signals are added to the primitive equation model through the outer boundaries which ignore the earth tides and astronomical tides potential flow [28]. The tidal force is computed using the TOPEX/Poseidon Global Inverse Solution (TPXO7.2) [29] with a resolution of 0.25° from the global barotropic tidal model of Oregon State University (refer to: http://volkov.oce.orst.edu/tides/global.html). Eight major tidal constituents (M_2_, S_2_, K_1_, O_1_, N_2_, K_2_, P_1,_ and Q_1_) are used. Their harmonic phases (in degrees) are presented in Universal Time (UT). The phase relation between Universal Time (UT) and Local Time (LT) is UT = LT—8ω, where the desired value of ω (in units of degrees/hour) is 28.984, 30.000, 15.041, and 13.943 for the M_2_, S_2_, K_1_, and O_1_ tides, respectively. Apart from the above, atmospheric and oceanic forces (such as wind stress, net heat and freshwater fluxes) from the Comprehensive Ocean-Atmosphere Data Set (COADS) [30], (refer to: http://iridl.ldeo.columbia.edu/SOURCES/.DASILVA/.SMD94/.climatology/) with a horizontal resolution of 0.5°×0.5° are also injected into the ROMS model. During the hourly integration of baroclinic tides model for the selected 90 days period (15^th^ December-15^th^ March), the model is initialized to the climatological monthly mean salinity [31] and temperature [32] fields from the World Ocean Atlas 2005 (WOA2005) (refer to: http://www.nodc.noaa.gov/OC5/WOA05/pubwoa05.html). Nevertheless, as the sea surface forces and values at the open boundaries vary with time, only the baroclinic processes are involved to give rise to different stratifications.

## Model Validation

From the last two months run for the inner domain of ROMS, *u*- and *v*-components as well as sea surface height are used to estimate the current and elevation for each tidal constituent based on the T-Tide harmonic analysis [33]. The estimates of simulated tidal phase and amplitude are compared with the tide gauges (TGs) data. Data from 19 TGs provided by the Department of Survey and Mapping Malaysia are used to validate the modelled sea surface elevation. Seventeen of these TGs are in the Malaysian waters, mostly distributed along the east coast and the southern end of Peninsular Malaysia, as well as Borneo. The remaining two gauges (Laut Island and Natuna Islands) are located within SSCS (Fig 1B). Tidal harmonic constants for these tidal gauges are computed and used for model validation.

The root mean square error (RMSE) differences in terms of amplitude and phase from global inverse tide model (TPXO7.2) and ROMS are computed with respect to those from tidal gauges (Table 1).

**Table 1:** RMSE differences in terms of amplitude and phase from global inverse tide model (TPXO7.2) and ROMS (outer and inner domains) computed with respect to those from tidal gauges.

	M_2_		S_2_		K_1_		O_1_	
	A.^a^	P.^b^	A.	P.	A.	P.	A.	P.
**TPXO7.2**	21	20	17	19	11	17	10	15
**ROMS: Outer domain**	19	16	12	17	8	16	6	13
**ROMS: Inner domain**	11	9	6	10	5	6	3	8

^a^ Amplitude (in cm)

^b^ Phase (in degrees) in local time, 8 hours after Universal Time (UT).

The RMSE for amplitudes between modelled and observed is computed using the following equation:

$$RMSE=\sqrt{\frac{\sum_{i=1}^{n}{\left|A_{mi}-A_{oi}\right|}^{2}}{n}}$$ (1)

**Fig 2:**
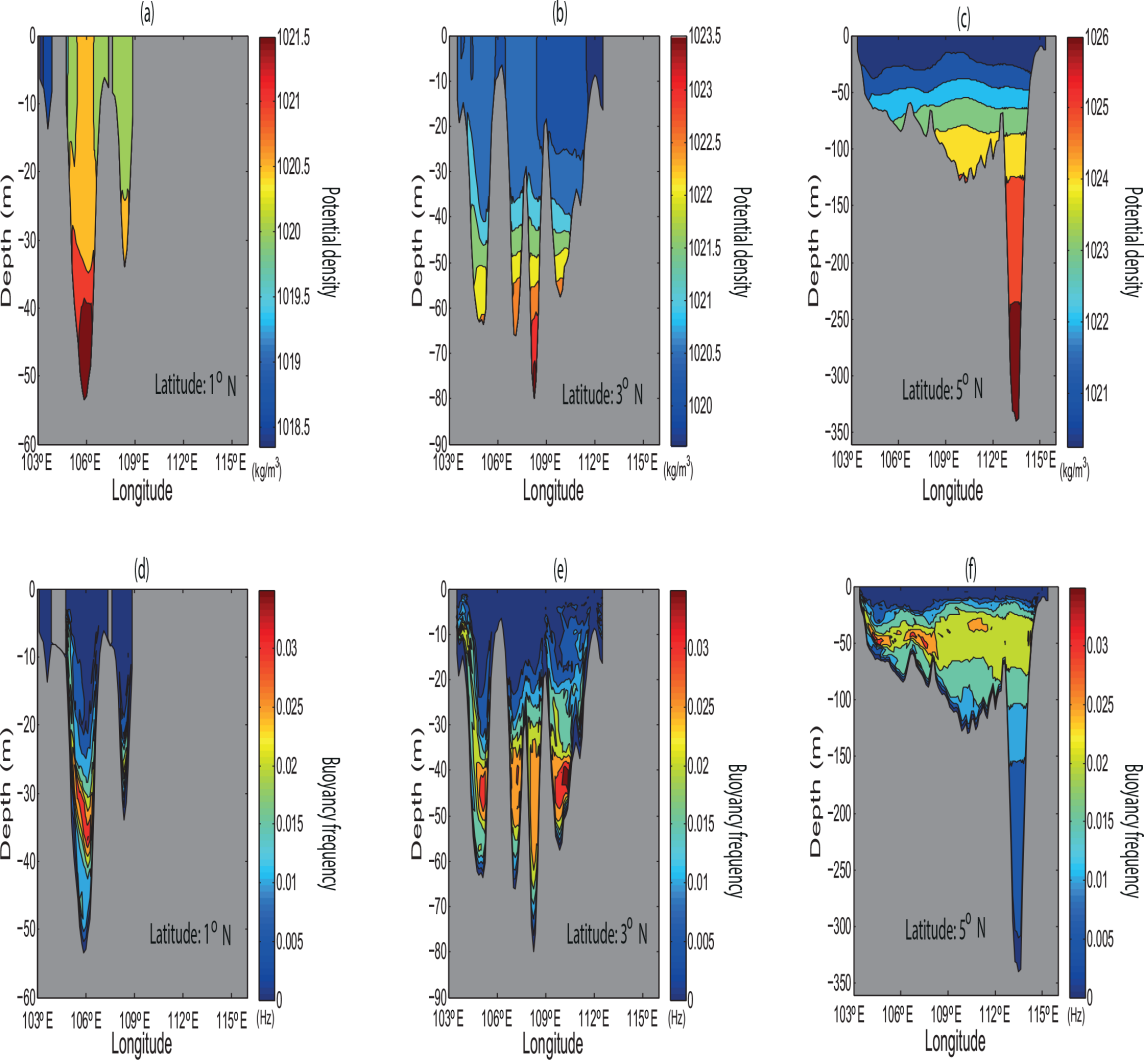
(*a-c*) Averaged annual potential density (kg/m^3^), and (*d-f*) buoyancy frequency (Hz) in different selected latitudes of the SSCS.

*A*_*mi*_ and *A*_*oi*_ are the respective modelled and observed amplitude at the station *i*. For the phases, the RMSE is computed by finding the pair-wise differences of the phases around the circle in degrees [34]. The inner domain of ROMS has the smallest RMSE in the estimated tidal amplitude and phase in comparison to those of the outer domain and TPXO7.2 (as evident in Table 1). SSCS is known to have complex bathymetry and coastlines. Complex bathymetry may lead to non-linear interaction between neighbouring tidal frequencies and baroclinic instabilities, which inject energy to the upper layers and modify the surface tidal signals [35]. As there are many small scale features in the region, higher resolution and smaller RMSE differences in amplitude and phase of the inner domain thus provide better simulation. More importantly, there is an additional need to accurately reproduce the stratification and baroclinicity of SSCS due to its error sensitivity towards bathymetry and model resolution. To attain this simulation, it can be demonstrated by analyzing the annual variations of average potential density and buoyancy frequency [6–7, 11] in the different selected latitudes of the SSCS with respect to the following equation (Eq 2) as shown in Fig 2.

$$N=\sqrt{-\frac{g}{\rho }\frac{\partial \rho (z)}{\partial z}}$$ (2)

where *N* is the Brunt–Väisälä frequency/buoyancy frequency (Hz), *g* is the acceleration of gravity (m/s^2^), and *ρ* the potential density (kg/m^3^). The stratification of layers and hence its indirect assessment of vertical displacement of water parcels are very clearly shown in Fig 2. Also, the existence of the maximum frequency of oscillatory fluid in the water column could not only lead to the formation and propagation of internal waves but also amplify the sea surface waves.

**Fig 3:**
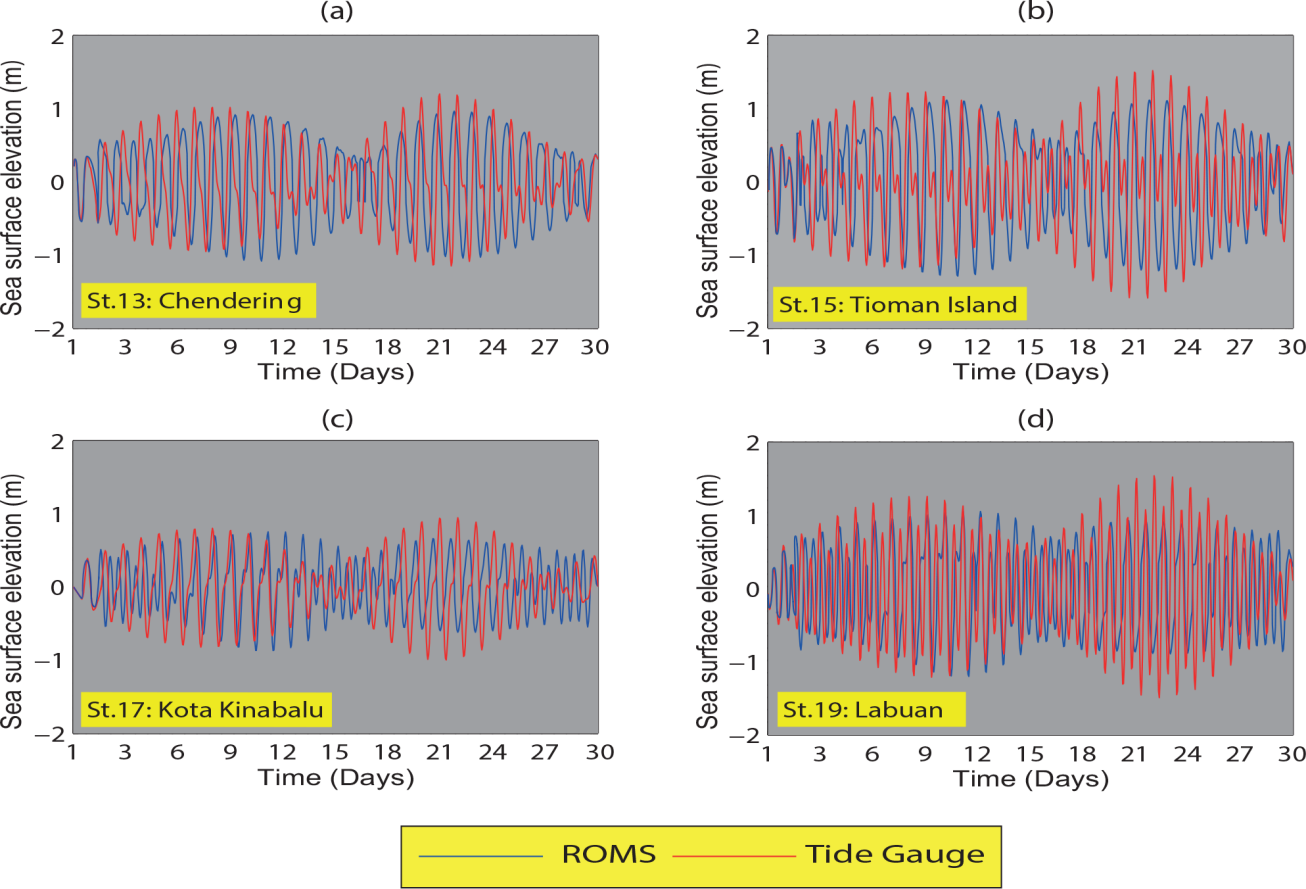
Observed (TGs) and simulated sea surface elevations (m) at (*a*) Chendering (Station 13), (*b*) Tioman Island (Station 15), (*c*) Kota Kinabalu (Station 17), and (*d*) Labuan (Station 19), averaged from January to March. The blue and red lines denote simulation and observation respectively.

The simulated and TGs elevations of the East Coast of the Peninsular Malaysia (ECPM) at Chendering, Tioman Island and the coastal regions of Sabah at Kota Kinabalu, and the Labuan station located off north-western Sabah are shown in Fig 3. The comparison between the TGs surface elevations and the simulated elevations shows that the spring-neap, diurnal and semidiurnal tides of the simulated model are reasonably reproduced by the model at all stations. This implies that the boundary conditions are accurately specified. However, small differences between the TGs and the simulated results can be expected due to the complex bathymetry [19, 35–36]. At the Labuan station, the elevation is simulated reasonably well in terms of phase and particularly, the tidal range. Results indicate that the largest tidal range occurs at Tioman Island (Station 15) and Labuan (Station 19), whereas the tidal ranges at Chedering (Station 13) and Kota Kinabalu (Station 17) are lower.

The skill of the model is assessed with a detailed comparison of the semidiurnal (M_2_ and S_2_) and diurnal (K_1_ and O_1_) tidal constituents for the simulated model and TGs as listed in Tables 2 and 3. Absolute error for the amplitudes and phases is calculated based on the differences between those of the model and those of the observed tides. Some stations indicate large absolute errors for both the amplitude and phase (see Tables 2 and 3). These large absolute errors may be due to the sensitivity of the tidal constituents to the bathymetry, especially in regions of steep bathymetry where the largest turbulent dissipation occurs [19, 35–36].

**Table 2:** M_2_ and K_1_ amplitudes and phases for the 19 stations of TGs and those simulated from the ROMS.

Station	Longitude	Latitude					M_2_							K_1_		
			Tide Gauge		ROMS		Absolute error		*d*^c^	Tide Gauge		ROMS		Absolute error		*d*
			A.^a^	P.^b^	A.	P.	DA.	DP.		A.	P.	A.	P.	DA.	DP.	
**1. Tumpat**	102.16° E	6.20° N	18	264	15	262	-3	-2	3	29	352	25	352	-4	0	4
**2. Terengganu**	103.13° E	5.35° N	27	244	22	243	-5	-1	5	46	8	46	5	0	-3	2
**3. Kuantan**	103.33° E	3.83° N	56	270	54	279	-2	+9	9	51	18	48	18	-3	0	3
**4. Laut Island**	108.00° E	4.75° N	9	56	10	54	+1	-2	1	36	333	35	336	-1	+3	2
**5. Natuna**	108.03° E	3.80° N	20	88	17	89	-3	+1	3	38	340	34	331	-4	-9	7
**6. Tanjong Datu**	109.65° E	2.08° N	91	117	90	117	-1	0	1	37	335	38	346	+1	+11	7
**7. Batang Mukah**	112.08° E	2.90° N	37	93	36	93	-1	0	1	40	326	41	340	+1	+14	10
**8. Miri**	113.60° E	4.28° N	16	342	17	342	+1	0	1	36	324	39	324	+3	0	3
**9. Kuching**	110.35° E	1.56° N	149	131	144	130	-5	-1	6	47	348	42	344	-5	-4	6
**10. Kuala Paloh**	111.23° E	2.45° N	110	114	109	115	-1	+1	2	46	338	44	344	-2	+6	5
**11. Johor Bahru**	103.75° E	1.46° N	87	325	40	326	-47	+1	47	30	103	40	101	+10	-2	10
**12. Tanjung Gelang**	103.43° E	3.96° N	53	261	47	258	-6	-3	7	53	13	59	19	+6	+6	8
**13. Chendering**	103.20° E	5.26° N	30	239	27	257	-3	+18	9	49	2	48	10	-1	+8	7
**14. Kukup**	103.41° E	1.31° N	92	326	100	296	+8	+30	50	26	164	30	173	+4	+9	6
**15. Tioman Island**	104.13° E	2.80° N	58	274	53	273	-5	-1	5	49	75	48	79	-1	+4	4
**16. Geting**	102.10° E	6.20° N	16	258	14	267	-2	+9	3	24	356	29	362	+5	+6	6
**17. Kota Kinabalu**	115.98° E	5.86° N	21	312	21	316	0	+4	1	30	313	36	320	+6	+7	7
**18. Bintulu**	113.03° E	3.16° N	16	44	19	51	+3	+7	4	47	320	53	330	+6	+10	11
**19. Labuan**	115.25° E	5.28° N	27	322	23	320	-4	-2	4	41	320	50	333	+9	+13	14
**Average absolute error**							-4	+4						+2	+4	

^a^ Amplitude (in cm)

^b^ Phase (in degrees) in local time, 8 hours after Universal Time (UT)

^c^ Difference (in cm) between the two sets of harmonic tides (observed and modelled) estimated as the distance in the complex plane.

**Table 3:** Same as Table 2, but for S_2_ and O_1_.

Station	Longitude	Latitude					S_2_							O_1_		
			Tide Gauge		ROMS		Absolute error		*d*	Tide Gauge		ROMS		Absolute error		*d*
			A.	P.	A.	P.	DA.	DP.		A.	P.	A.	P.	DA.	DP.	
**1. Tumpat**	102.16° E	6.20° N	8	295	9	303	+1	+8	2	17	316	17	309	0	-7	2
**2. Terengganu**	103.13° E	5.35° N	11	288	13	283	+2	-5	2	29	320	28	313	-1	-7	4
**3. Kuantan**	103.33° E	3.83° N	17	322	16	312	-1	-10	3	35	334	32	336	-3	+2	3
**4. Laut Island**	108.00° E	4.75° N	4	56	3	54	-1	-2	1	18	268	11	264	-7	-4	7
**5. Natuna**	108.03° E	3.80° N	7	128	6	130	-1	+2	1	28	311	27	318	-1	+7	4
**6. Tanjong Datu**	109.65° E	2.08° N	18	141	19	149	+1	+8	3	15	256	16	254	+1	-2	1
**7. Batang Mukah**	112.08° E	2.90° N	9	107	6	100	-3	-7	3	34	268	32	272	-2	+4	3
**8. Miri**	113.60° E	4.28° N	8	22	8	22	0	0	0	31	271	29	276	-2	+5	3
**9. Kuching**	110.35° E	1.56° N	45	189	43	192	-2	+3	3	34	293	33	300	-1	+7	4
**10. Kuala Paloh**	111.23° E	2.45° N	30	176	35	182	+5	+6	6	37	288	36	284	-1	-4	3
**11. Johor Bahru**	103.75° E	1.46° N	34	23	10	20	-24	-3	24	30	50	27	49	-3	-1	3
**12. Tanjung Gelang**	103.43° E	3.96° N	18	309	18	294	0	-15	5	36	329	35	310	-1	-19	12
**13. Chendering**	103.20° E	5.26° N	12	283	12	285	0	+2	0	30	316	34	312	+4	-4	5
**14. Kukup**	103.41° E	1.31° N	42	19	45	10	+3	-9	7	25	119	25	113	0	-6	3
**15. Tioman Island**	104.13° E	2.80° N	18	327	19	353	+1	+26	8	34	343	37	350	+3	+7	5
**16. Geting**	102.10° E	6.20° N	8	298	10	282	+2	-16	3	13	303	9	305	-4	+2	4
**17. Kota Kinabalu**	115.98° E	5.86° N	11	353	9	358	-2	+5	2	30	263	30	252	0	-11	6
**18. Bintulu**	113.03° E	3.16° N	6	32	6	33	0	+1	0	32	271	31	279	-1	+8	5
**19. Labuan**	115.25° E	5.28° N	12	9	10	5	-2	-4	2	33	262	29	270	+4	+8	6
**Average absolute error**							-1	+1						-1	-1	

The *d* value which is the difference between the two sets of harmonic tides (observed and modelled) estimated as the distance in the complex plane according to the following formula [28],

d={(Aocos⁡po−Amcos⁡pm)2+(Aosin⁡po−Amsin⁡pm)2}12 (3)

where *A*_*o*_, *A*_*m*_, *p*_*o*_, and *p*_*m*_ represent the observed and modelled amplitudes and phases, respectively. The obvious differences for M_2_ occurred at Johor Bahru (Station 11) with 47 cm and at Kukup (Station 14) with 50 cm, both at the southern mouth of the Strait of Malacca. Such difference can be attributed to the insufficient model resolution, leading to the inaccurate representation on the dissipation of energy caused by bottom friction and baroclinicity [28]. Also, the tidal waves response from the model may be very sensitive to the local bathymetry and coastline.

**Fig 4:**
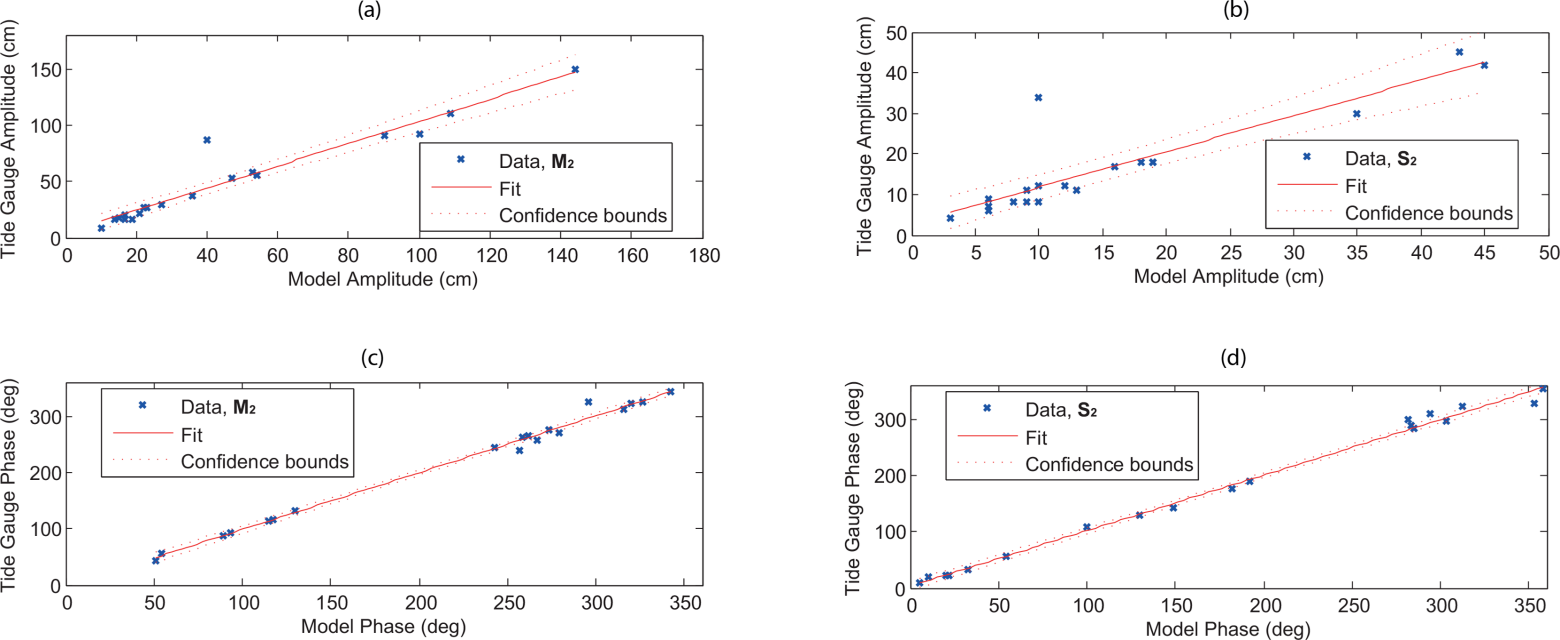
Linear regression with 95% confidence intervals for (*a*-*b*) tidal amplitude (cm) and (*c*-*d*) phase (in LT degrees) of semidiurnal tidal constituents (M_2_ and S_2_) between 19 tide gauges and estimated from the simulated model.

**Fig 5:**
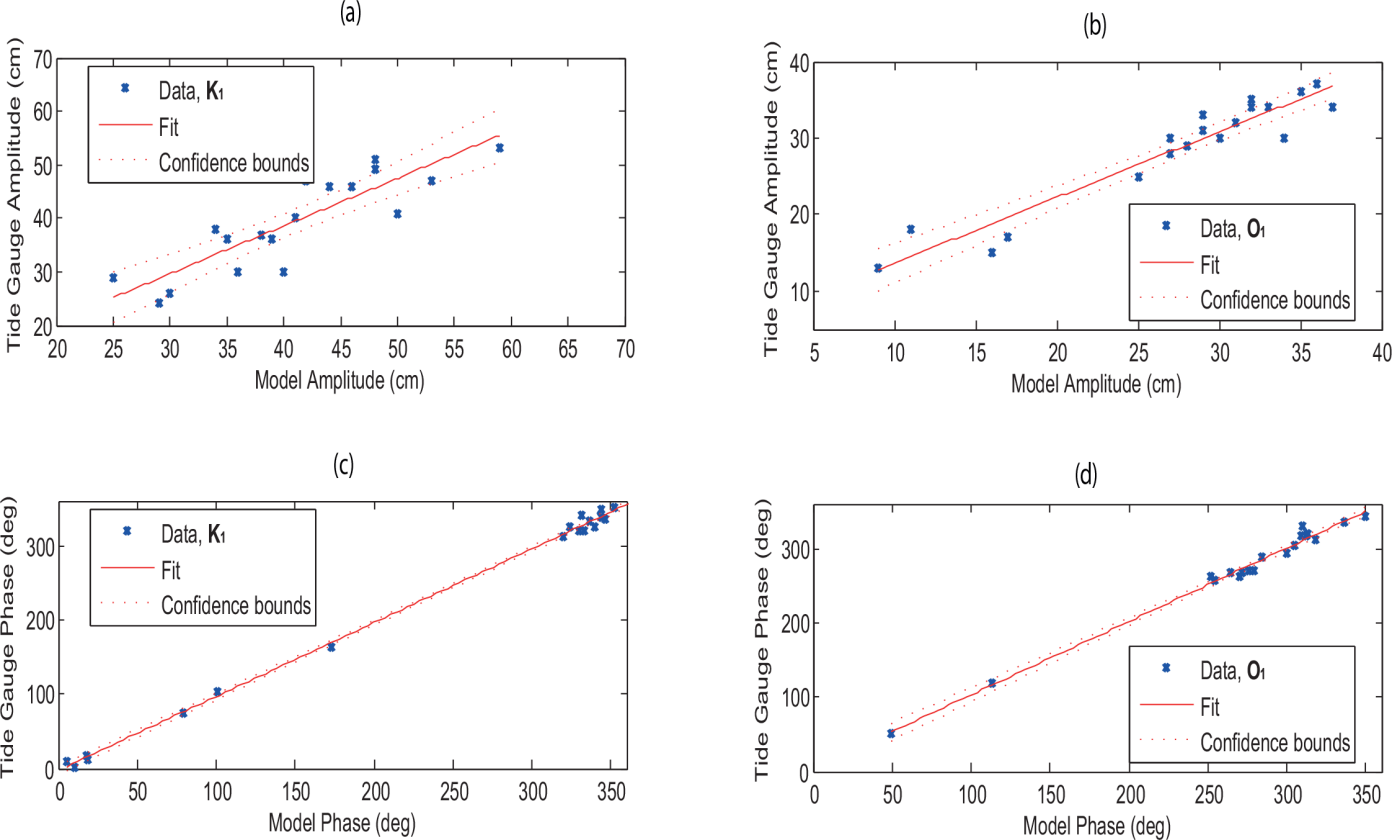
Same as Fig 2, but for diurnal tidal constituents (K_1_ and O_1_).

The estimated amplitudes and phases from the simulated model for the semidiurnal (M_2_ and S_2_) and diurnal (K_1_ and O_1_) tidal constituents are compared against the corresponding values observed from the TGs (Figs 4 and 5). The estimation is done using the following linear regression equation with 95% confidence intervals,

$$\left\{ \begin{array}{l} \;y_{i}={\hat{\beta }}_{0}+{\hat{\beta }}_{1}x_{i}+e_{i}\\ {\hat{\beta }}_{1}=\frac{\sum_{i=1}^{n}\left(x_{i}-\overset{-}{x})(y_{i}-\overset{-}{y}\right)}{\sum_{i=1}^{n}{\left(x_{i}-\overset{-}{x}\right)}^{2}}\;and\;{\hat{\beta }}_{0}=\overset{-}{y}-{\hat{\beta }}_{1}\overset{-}{x} \end{array}\right.$$ (4)

where *n* represents the number of stations, *x*_*i*_ and *y*_*i*_ are the model and observed variables at the station *i*. The regression coefficients of *β*_0_ and *β*_1_ are known as the y-intercept and the slope respectively, and $e_{i}=y_{i}-{\hat{y}}_{i}$ is the error term. It can be seen that the estimated semidiurnal and diurnal tidal phases and amplitudes match reasonably with the observed values as these values lie between the confidence bounds (Figs 4 and 5). There are only two points which are significantly away from the confidence bound for semidiurnal (M_2_ and S_2_) tides. These points occurred at Johor Bahru (Station 11) with 47 and 24 cm absolute error (as evident in Tables 2 and 3).

## Basic Tidal Features

### M_2_ tide

**Fig 6:**
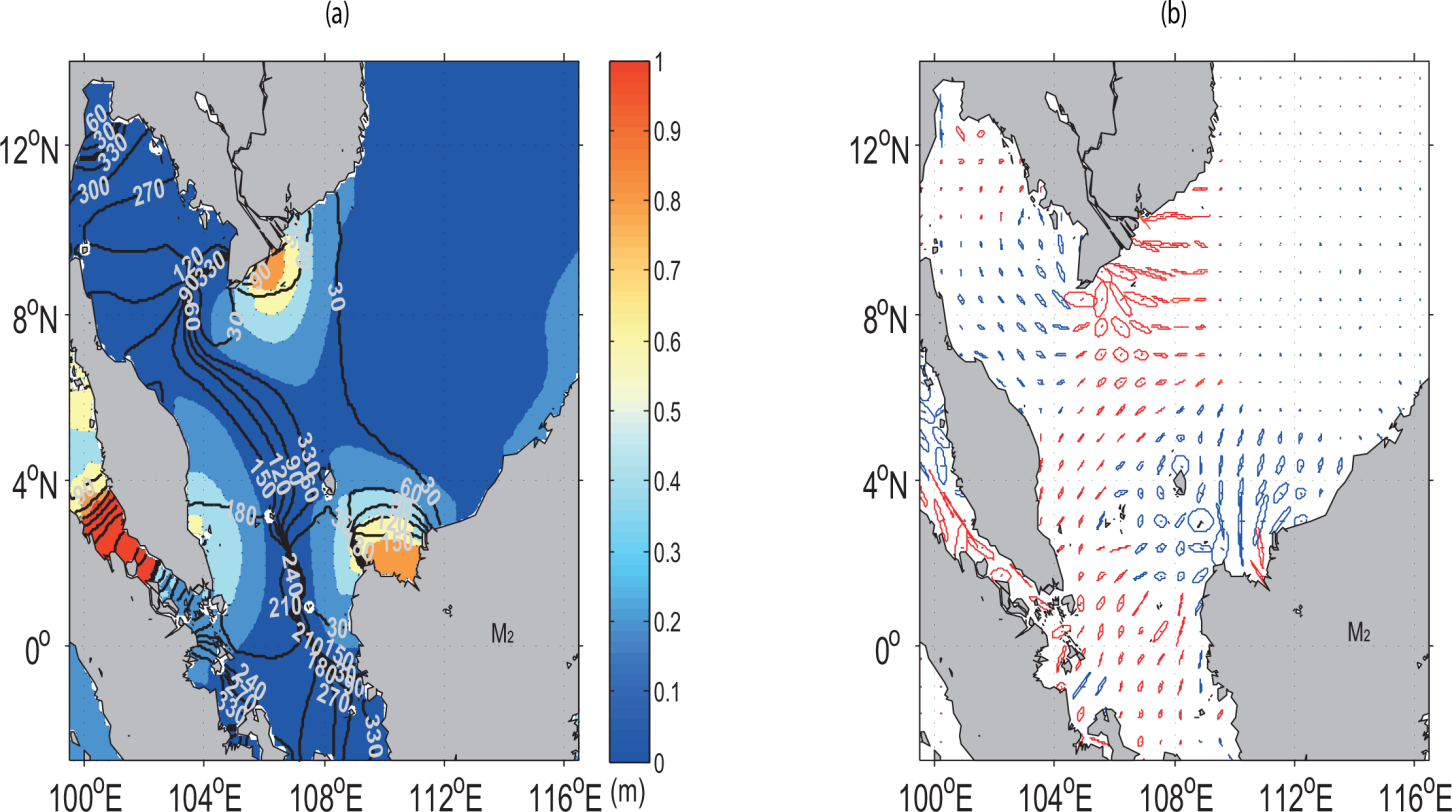
Contours in (*a*) denote the co-phase lines (in UT degrees) for the M_2_ constituent superimposed by the magnitude of the co-amplitude (m, colour shaded), and (*b*) shows the tidal current ellipse (counter clockwise rotation in red and clockwise rotation in blue). The maximum current with the largest major semi-axis is 0.7 m/s.

The spatial pattern and magnitude of amplitude and phase lags from the simulated M_2_ tide are generally similar to those found by Fang *et al*. [17], Zu *et al*. [18] and Green and David [19] (Fig 6A).

The M_2_ amplitude is generally higher (> 0.7 m) along the southern Vietnam coast and the north-western coast of East Malaysia due to the refraction of the waves from the coastlines. For the ECPM it is approximately 0.5 m higher in the southern coast and lower in other areas as a consequence of strong shoaling and narrowing effects from the deep basin [18].

Based on the works of Fang *et al*. [17], a simulated elongated nodal band is found to originate from the southern tip of the Gulf of Thailand, running parallel to the ECPM. This node ends somewhere between the southern Peninsular Malaysia and northwest coast of East Malaysia. Along this nodal band, there exits two amphidromic points located at 8.5° N, 104.5° E and 2° N, 108° E, respectively, with clockwise rotation. The simulated amphidromic point at 8.5° N, 104.5° E is noted to be almost consistent with those done by Yanagi and Takao [37], Fang *et al*. [17], Jan *et al*. [38] and Zu *et al*. [18]. However, the amphidormic point at 2° N and 108° E is not featured in either Fang *et al*. [17] and Zu *et al*. [18]. The existence of amphidormic points in shallow waters is due to the fact that the bottom stress is computed using the velocity of the layer nearest to the seabed [39–41] in the three dimensional model instead of using uniform mean velocity as in the two dimensional model.

The simulation shows that the tidal waves from the deep basin reach the Sunda shelf in the form of standing waves. Propagation speed, *C* (m/s), is estimated by the following equation:

C=L(Δg360°)×TM2 (5)

where *L* denotes the distance between two neighbouring co-phase lines, and (Δg360°)×TM2 is the time needed for the waves to travel across this distance. Δ*g* is the difference between two neighbouring co-phase lines in degrees and $T_{M_{2}}$ the period of the M_2_ tide. Using the values between 30° and 330° of the co-phase lines (Fig 6A) in the Sunda Shelf, Δ*g* = 300°, $T_{M_{2}}=12.4$ hours, and *L* ≅ 445 km, *C* in the continental shelf area is found to be ~12 m/s. The wave speed for the M_2_ in the SCS deep basin is approximately 164 m/s [18]. This implies that the wave propagation in the deep basin is faster than that in the continental shelf. Apart from the wave speed, the tidal currents are strong in the continental shelf but weak in the deep basin (as evident in Fig 6B). Furthermore, the large and strong currents coincide with the areas of high amplitudes.

### K_1_ Tide

**Fig 7:**
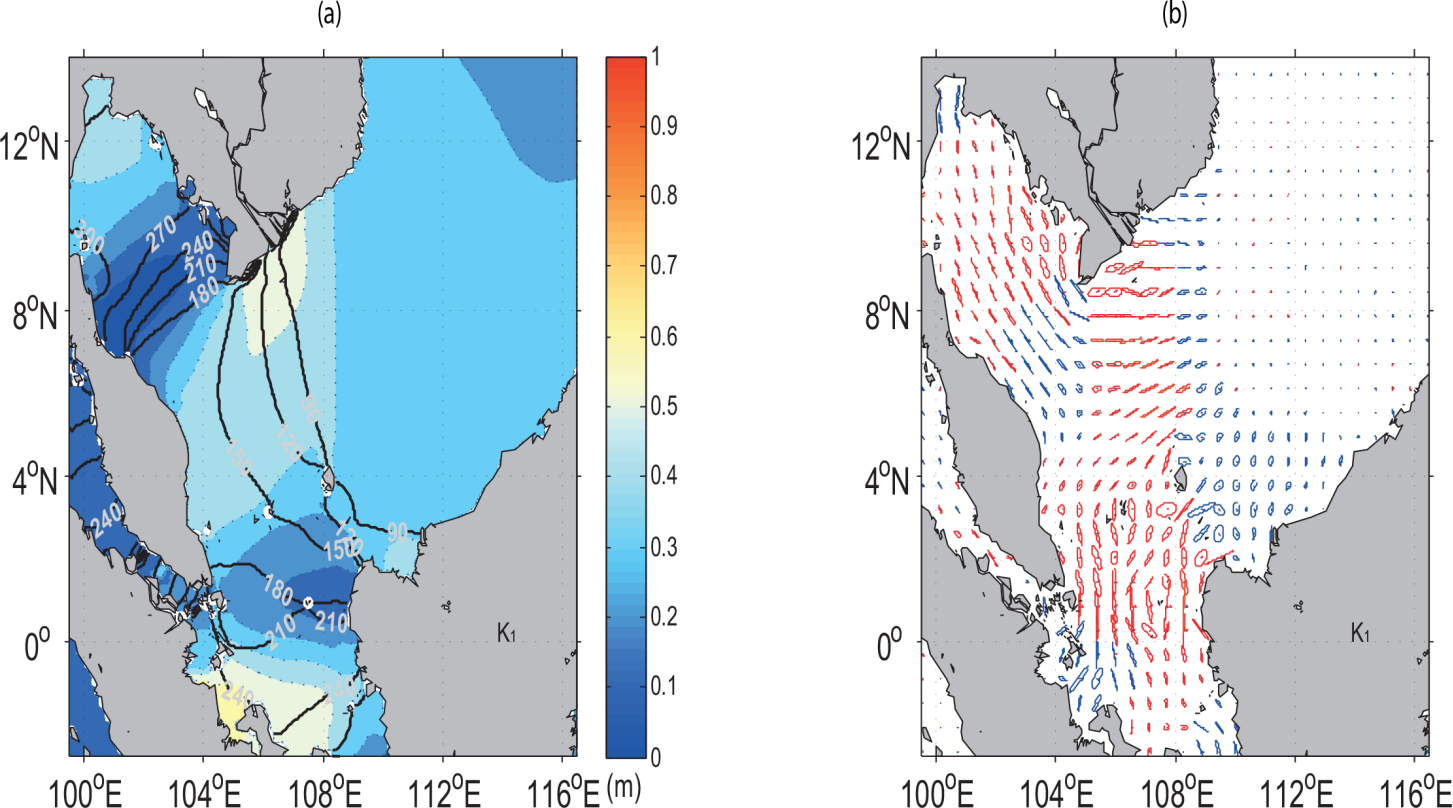
Same as Fig 6, but for the K_1_ constituent, the maximum current with the largest major semi-axis is 0.43 m/s.

The spatial pattern of the K_1_ amplitudes is much different from that of the M_2_ even though it shows similar tendency of having higher values over the continental shelf than in the deep open sea region (Fig 7A).

In comparison with the M_2_ tide, two semi-circular cores of high tidal amplitudes are no longer visible off south-eastern Vietnam and western East Malaysia. High K_1_ amplitudes are instead found closer to southern Sumatra in the Karimata Strait as shown in Fig 7A. As the tidal currents propagate from the deep basin into the continental shelf, they diverge partly into the Gulf of Thailand and turn counter-clockwise in contrast with that of M_2_. The contrast in rotation here may due to its specific tidal phase [37]. The tidal currents also flow southwards into the Java Sea through the Karimata Strait in similar counter-clockwise direction (Fig 7B). In general, the M_2_ co-phase lines are densely clustered with smaller lines as compared with those of the K_1_ tidal waves.

### S_2_ and O_1_ Tides

**Fig 8:**
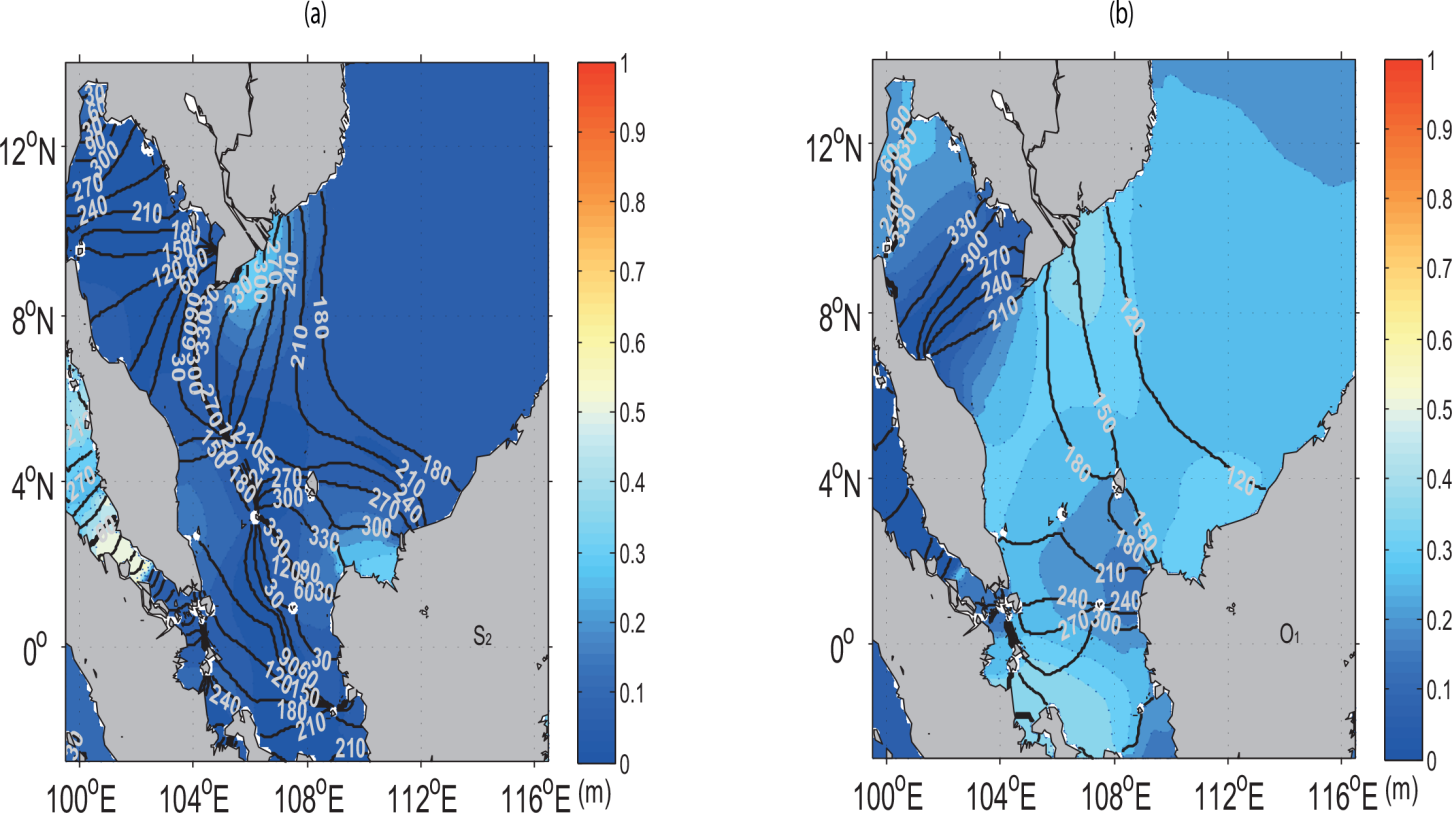
Contours in (*a*) and (*b*) respectively denote the S_2_ and O_1_ co-phase lines (in UT degrees) superimposed by the magnitude of the co-amplitude (m, colour shaded).

The characteristics of two tidal constituents, the principal solar semidiurnal (S_2_) and lunar diurnal (O_1_) are shown in Fig 8. Similar co-amplitude and co-phase patterns in M_2_ and K_1_ are also noted in S_2_ and O_1_. In terms of amplitude, the S_2_ is smaller than the M_2_ while the O_1_ is smaller than the K_1_. These results are consistent to those obtained by previous studies [17–18, 42]. Only four amphidromic points can be seen in the S_2_ at 9.5° N, 105° E; 5° N, 105° E; 3° N, 106.5° E and 1.7° S, 109° E. These points are consistent with those obtained in Mao *et al*. [42].

## Tidal Dynamics

### Tidal resonance

Tidal resonance occurs when the tidal force excites one of the resonant modes of the coastal sea, especially when the continental shelf width is of a quarter wavelength. Consequently, tidal energy strengthens due to reflections between the coast and the vicinity of the shelf edge, leading to the production of a very high tidal range at the coast. Assuming that the tidal wave length from the mouth of the SSCS (see Fig 1B, dashed line (T1) with the length of *x* between L0-L1), i.e., the head of the channel which is the pathway between the mouth of SSCS and the Java Sea is much larger than the mean depth of the channel, shallow water equations are therefore applicable to simulate the tidal waves as described in the following equation:

$$\left\{ \begin{array}{c} \frac{\partial u}{\partial t}=-g\frac{\partial \eta }{\partial x}\\ \frac{\partial \eta }{\partial t}=-h\frac{\partial u}{\partial x} \end{array}\right.$$ (6)

where *η* (m) is the elevation, and *u* (m/s), the zonal component of the current along the T1. By setting *u* = 0 along the T1 at all times, the solution of Eq (6) is in the form of the standing tidal wave shown in the following equation,

$$\left\{ \begin{array}{l} u=u_{max}\;\sin(kx)\;\sin(\sigma t)\\ \;\eta =A\;\cos(kx)\;\cos\left(\sigma t\right) \end{array}\right.$$ (7)

where *u*_*max*_ is the maximum tidal current, $A=\frac{\sigma u_{max}}{gk}$ is the wave amplitude, $k=\frac{2\pi }{\lambda }$ is the wave number, $\sigma =\frac{2\pi }{T\lambda }$ is the wave frequency, *T* and *λ* are the wave period and wave length, respectively. In terms of the corresponding wave dispersion relation (*σ*^2^ = *ghk*^2^), the wave length (λ) is $\sqrt{gh}$. If the tidal amplitude at the head of the channel (*x* = *L*_*0*_*L*) is *A*_*x*,_ then according to a study by Bowden [43], the elevation and amplitude along the T1 can be written as *η* = *A*_*x*_ cos(*σt*) and *A* = *A*_*x*_/cos(*kx*), respectively.

Resonance will occur if cos(*kx*) ≅ 0, with the arguments of cosine ($kx=\frac{\pi }{2},\frac{3\pi }{2},\frac{5\pi }{2},\ldots ,\frac{(2n-1)\pi }{2}$, *n* = 1, 2,…). The first resonance mode ($n=1\to kx=\frac{\pi }{2}$) with the length *x* $=\frac{\lambda_{itw}}{4}$ leads to the Helmholtz resonance in which *λ*_*itw*_ is defined as the length of the incoming tidal wave. Nevertheless, a computational estimation based on the following Eq (8) which is associated with the resonant angular frequency (*ω*_*0*_) and period (*T*_*0*_), by assuming the length *x* = *L*_*0*_*L* ≅ 500 km and average depth of the channel *h* ≅ 50 m at the entrance of the channel, the resonance period in the interface between the channel and the basin (SSCS) is approximately 25 hours.

$$\left\{ \begin{array}{c} \omega_{0}=2\pi \frac{\sqrt{gh}}{4x}\\ T_{0}=4\pi \frac{1}{\sqrt{gh}} \end{array}\right.$$ (8)

The above estimated value is approximately equal to the periods of K_1_ (23.93 hours) and O_1_ (25.82 hours) tidal constituents. This implies that the amplitude of the diurnal tides increases similar to the Helmholtz resonance after the tidal wave propagates from the SSCS into the Java Sea through the Karimata Strait.

### Tidal energy flux and its dissipation

**Fig 9:**
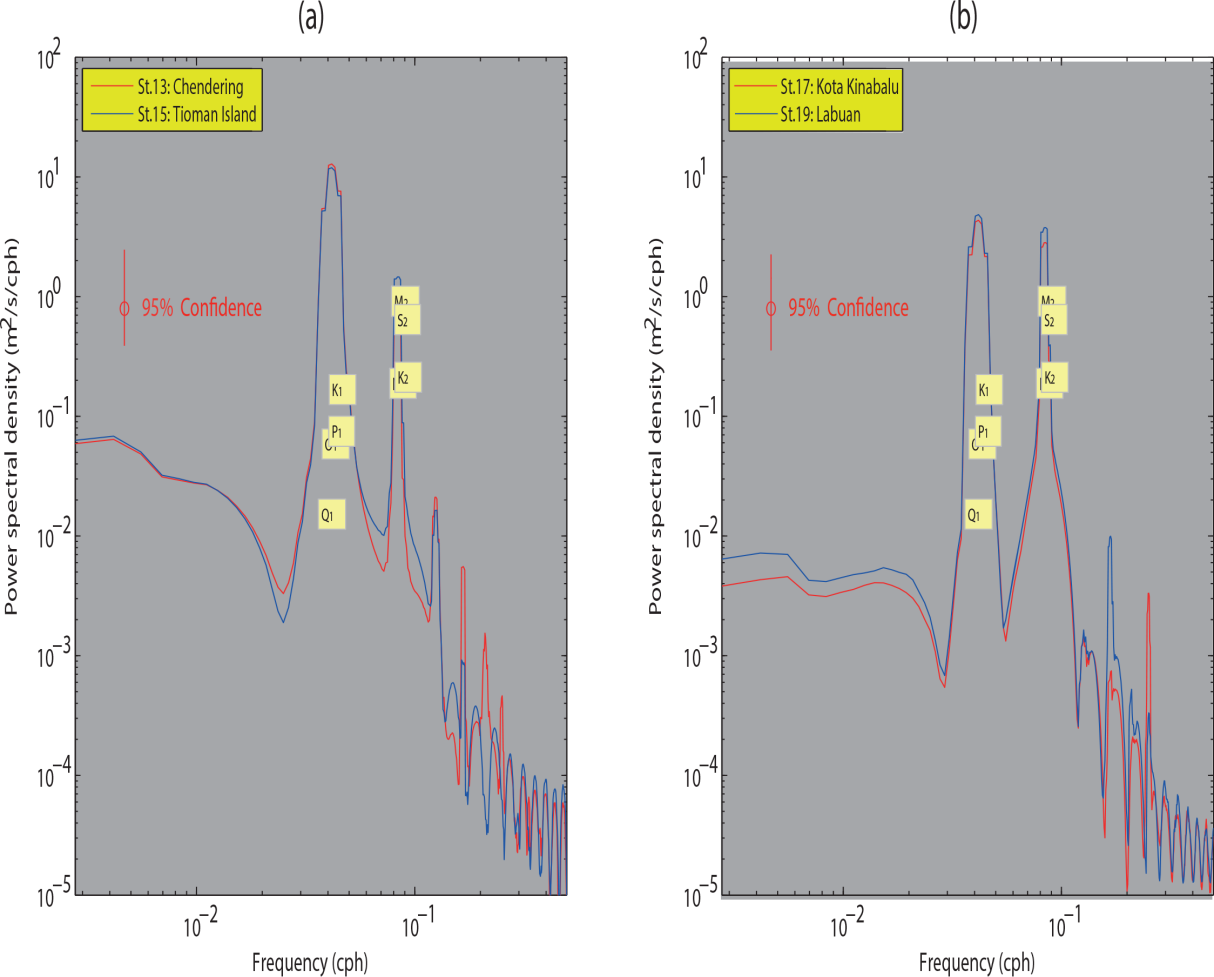
Power spectral density (m^2^/s/cph) estimated for elevations at (*a*) Chendering and Tioman Island, and (*b*) Kota Kinabalu and Labuan stations.

The strength of the tidal constituents’ variations (energy) in the continental shelf of the SSCS is assessed using power spectral density function (m^2^/s/cph). It shows that the diurnal frequencies are most dominant at the selected stations (as evident in Fig 9). The mean power energy for diurnal tides at Labuan (Station 19) is approximately 43% less than that at Chendering (Station 13), while the mean power energy for the semidiurnal tides at Labuan (Station 19) is greater by approximately 25% than that at Chendering (Station 13). This implies that the diurnal tide is more energetic along the ECPM, while both diurnal and semidiurnal tides are almost equally energetic at coastal East Malaysia.

To assess the tides and its energy dissipation in the continental shelf of the SSCS, the tidal energy flux (*J* = W/m) is computed based on the following equation:

$$\left\{ \begin{array}{l} E=\frac{1}{2}\rho_{0}h(u^{2}+v^{2})\\ \;J=EC_{g} \end{array}\right.$$ (9)

where *E* is the total tidal energy per unit area, *ρ*_0_(≅1025 kg/m^3^) is the mean density of the seawater, *h*, the depth of the water column (m), *u* and *v* (m/s) are the vertically averaged zonal (semi-major) and meridional (semi-minor) components of the tidal currents, respectively. The group velocity *C*_*g*_ (m/s) is given by;

$$C_{g}=\frac{C}{2}\left(1+\frac{2kh}{sinh(2kh)}\right)$$ (10)

where $C=\sqrt{gh}$ is the wave celerity in m/s, $k=\frac{2\pi }{\lambda }$, the wave number, *λ = TC* denotes the wave length (m), and *T* (in sec) is the period for each tidal constituent.

**Fig 10:**
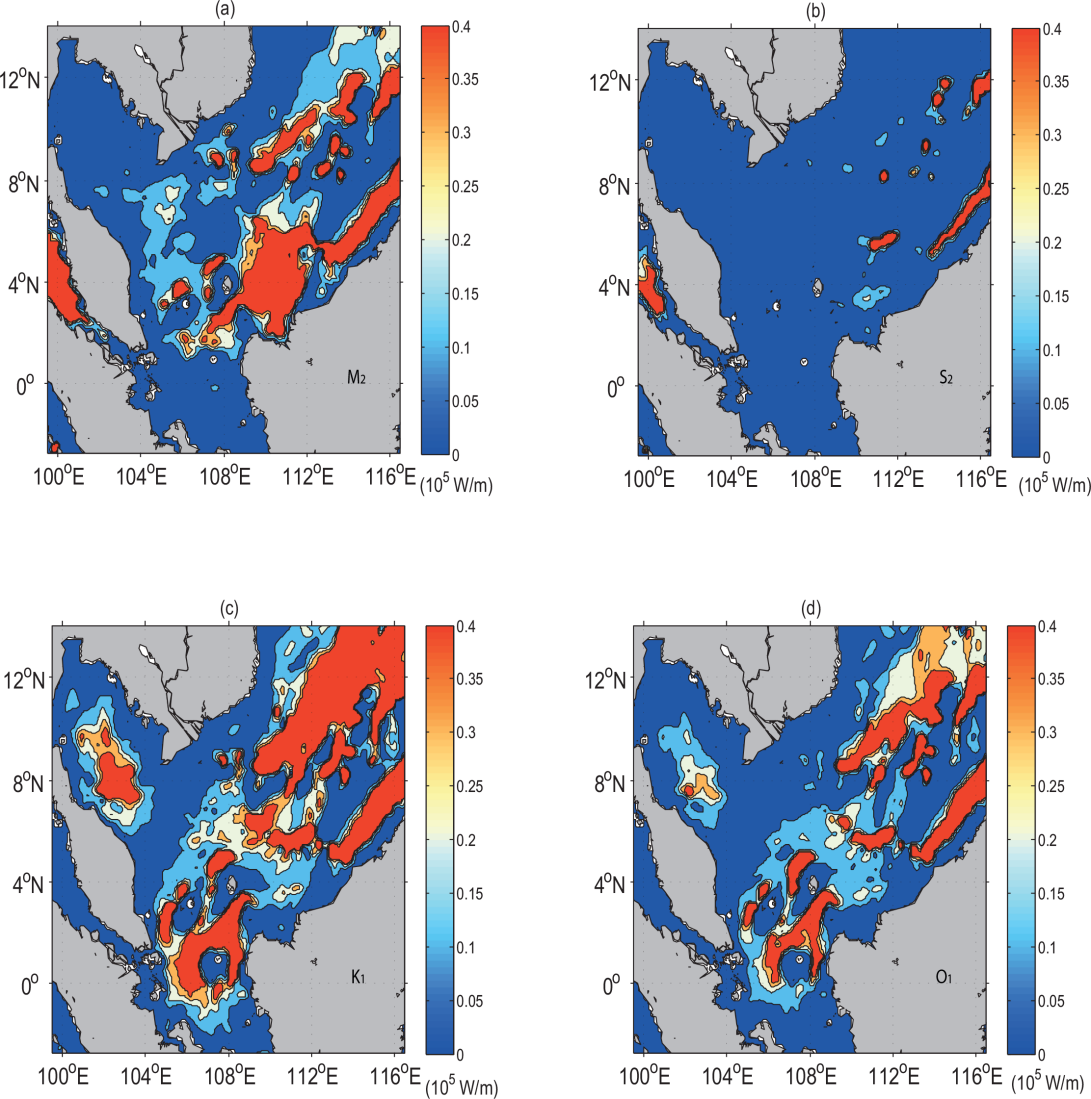
Tidal energy flux (10^5^ W/m), (*a*-*b*) semidiurnal tides (M_2_ and S_2_) and (*c*-*d*) diurnal tides (K_1_ and O_1_).

The general patterns of the tidal energy flux for the diurnal tides (the K_1_ and O_1_ constituents) are dominant as compared with the semidiurnal tides over the continental shelf of the SSCS (Fig 10). The high semidiurnal tidal energy flux of approximately 0.4×10^5^ W/m is seen near the coastal regions of Sabah and Sarawak, especially near Tanjung Datu (Station 6), Kuching (Station 9) and Kuala Paloh (Station 10) which are located in the coastal areas of Sarawak (Fig 10A). This indicates that these areas are primarily influenced by the semidiurnal tides. Similarly, the strong tidal energy flux of both the diurnal and semidiurnal tides are found stretching along the coastal areas of Sabah (station Kota Kinabalu), Sarawak (station Miri), and Labuan. However, it was noted that the dissipation of the tidal waves with strong tidal energy flux of approximately 0.4×10^5^ W/m tends to be at Miri (Station 8) located at the coast of Sarawak.

The diurnal tidal energy flux dissipates in the Java Sea after traversing the SSCS through the Karimata Strait (Fig 10C and 10D). Here, the mean diurnal tidal energy flux is 6% greater than the semidiurnal tides in the area between 105° E-109° E and 5° S-1° N. In general, the total mean diurnal tidal energy flux over the whole domain between 103° E-116° E and 5° S-8° N is 60% greater than that of the semidiurnal tides, implying the dominance of the diurnal tides in the region. These results are in good agreement with those previous studies which have noted that the dissipation of tides from the Pacific Ocean into the SCS is primarily diurnal [44–45].

## Tidal Mixing Fronts

The mixing parameter (given by log10(hU3)) from Simpson and Hunter [46] is used to assess the contribution of tidal waves in shallow water on key biological processes that lead to the transport of nutrients and phytoplankton blooms across a tidal mixing fronts, where *h* (m), is the water depth and *U*, the tidal current amplitude (m/s).Yanagi *et al*. [47] shows the existence of the tidal mixing fronts in the SSCS for the northern Gulf of Thailand and the offshore area of the ECPM. Previous findings have pointed out that the regions with smaller parameter values of ~<3 are locations of tidal mixing fronts [47–49]. Hence, coastal areas of Sarawak, namely Tanjung Datu (Station 6), Kuching (Station 9), and Kuala Paloh (Station 10), are found to be the locations of tidal mixing fronts due to the existence of strong M_2_ tide because of its maximum tidal current and strong tidal energy flux. For the diurnal tides, the maximum tidal current is clearly correlated with the strong energy flux in the region between southern tip of the ECPM and western East Malaysia (Fig 10C and 10D).

**Fig 11:**
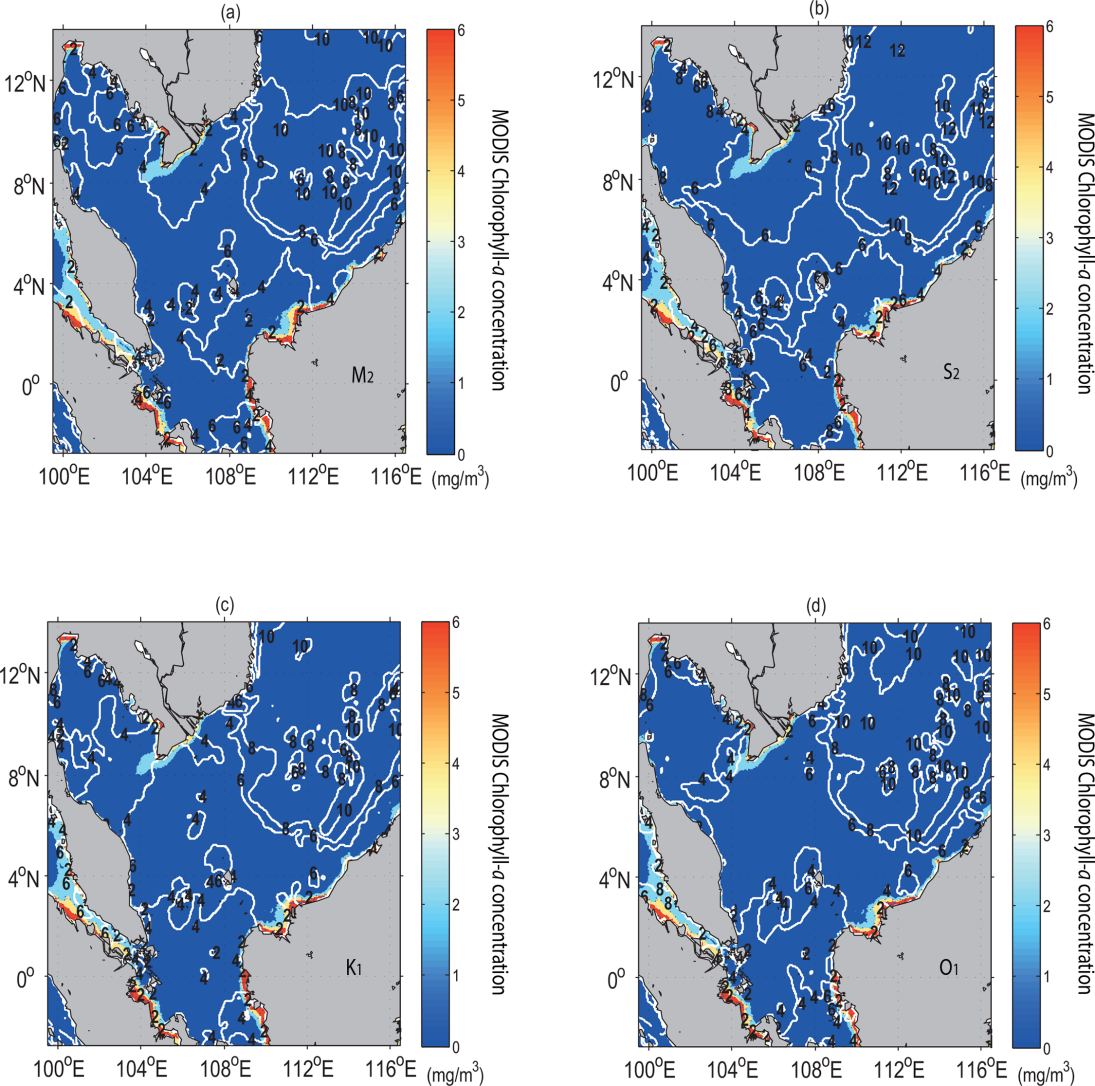
Simulated tidal mixing fronts (in contours), (*a*-*b*) semidiurnal tides (M_2_ and S_2_), and (*c*-*d*) diurnal tides (K_1_ and O_1_) superimposed by distribution of the bi-monthly (January-February) MODIS chlorophyll-*a* concentration (mg/m^3^, colour shaded).

To distinguish the effect of tidal mixing from the other atmospheric and oceanic forces, the bi-monthly (January-February) averages of the concentration of chlorophyll-*a* (in mg/m^3^) from the Moderate Resolution Imaging Spectroradiometer (MODIS) Aqua for the periods 2005–2013 (http://podaac.jpl.nasa.gov/dataset/MODIS_Aqua_L3_CHLA_Daily_4km_R), which is an appropriate proxy of phytoplankton biomass along the coastline is used to assess its response to the various corresponding forces. It is found that areas with mixing parameter values ~< 3 are well correlated with the areas of strong tidal current and high tidal energy flux which are supported by the high concentrations of chlorophyll (as evident in Fig 11).

**Fig 12:**
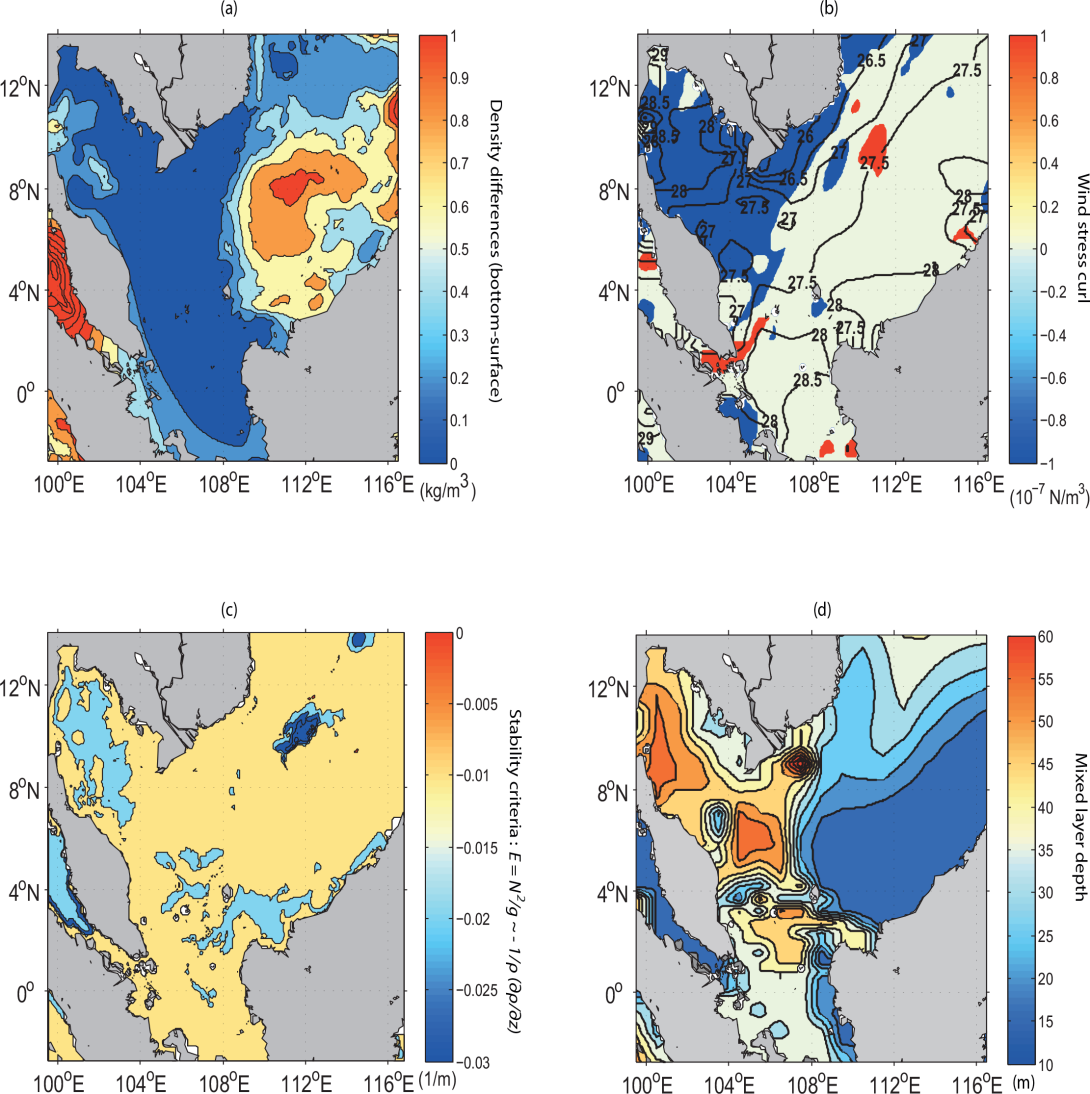
Bi-monthly surface distribution for (*a*) density difference (kg/m^3^) between the sea surface and its subsurface, (*b*) the potential temperature (in contour, °C) superimposed by wind stress curl (10^−7^ N/m^3^), whilst (*c*) and (*d*), respectively, indicate stability criteria and mixed layer depth (m).

**Fig 13:**
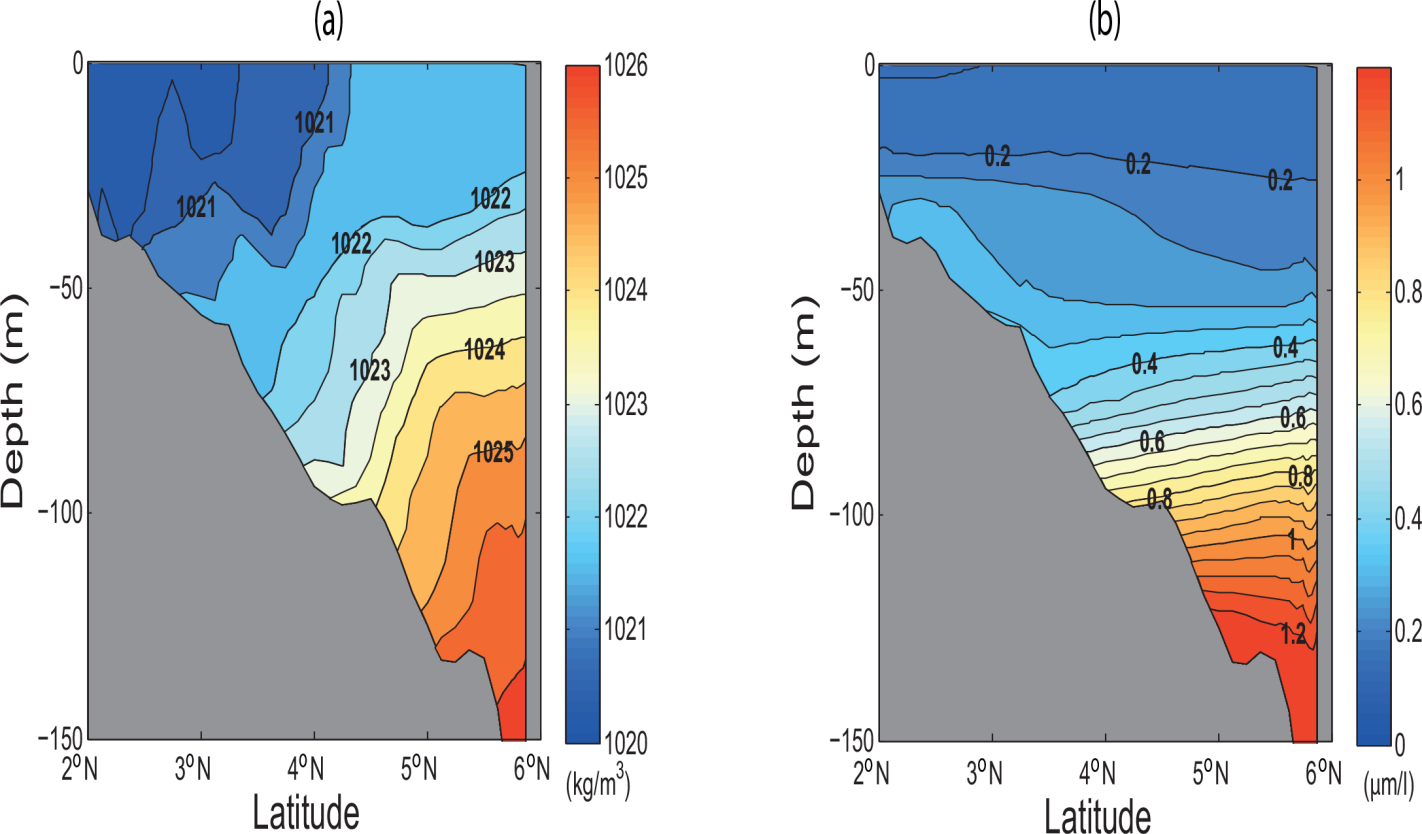
Bi-monthly profiles along transect A1A2 (see Fig 1B, the location of transect) for (*a*) simulated density (kg/m^3^) and (*b*) observed phosphate (μm/l) derived from WOA2005.

**Fig 14:**
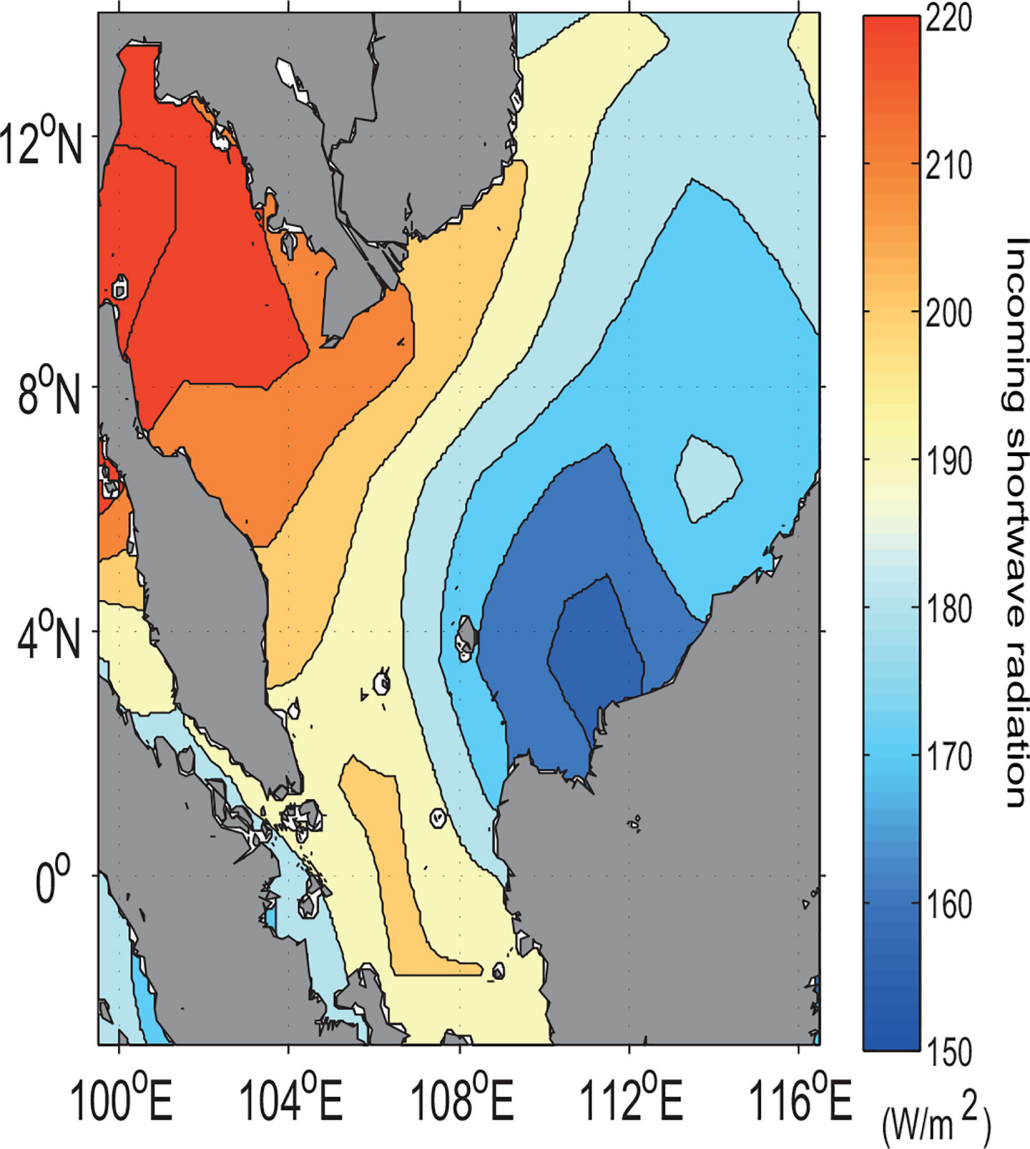
Bi-monthly sea surface distribution of incoming shortwave radiation (W/m^2^) derived from ECMWF reanalysis data (refer to: http://apdrc.soest.hawaii.edu/datadoc/ecmwf_oras3.php).

Tidal mixing processes for this region can be assessed from the distributions not only of the bi-monthly wind stress curl (derived from COADS [30]) and simulated density difference between the sea surface and its subsurface, but also of the potential temperature ($\theta =T+\int_{P}^{P_{0}}\Gamma (S,T,P)dP$ which is a function of Pressure (*P*), where Γ is adiabatic lapse rate, *S* and *T* are salinty and temperature, respectively) as computed from the model. Accordingly, both low values of density difference and potential temperature upon the area of zero curl (implying no Ekman transport) (Fig 12A and 12B) in the vicinity of coastal Sarawak indicate that this area is well mixed. Hence, the buoyant tidal fronts become significant and enhance the instability (see Fig 12C, given by $E=\frac{N^{2}}{g}∼\frac{-1}{\rho }\frac{\partial \rho }{\partial z}$). This causes the cold water at the sea surface to induce vertical convection and subsequent mixing to a relatively deep mixed layer depth (as evident in Fig 12D) based on calcualtion by Lorbacher *et al*. [50]. The density profile along the transect A1A2 (see Fig 13A) reveals that denser water is uplifted to the sea surface in conjunction with the well-mixed and uniform concentration of nutrients (such as phosphate) from the sea surface to an approximate depth of 25 m (see Fig 13B). It is also to be noted that this area has low incoming shortwave radiation (Fig 14). Therefore, the high chlorophyll concentration at the sea surface in the vicinity of coastal Sarawak is the outcome of the M_2_ tidal mixing front generated by the high tidal energy flux there. In contrast, along the western coast of Borneo, the high incoming shortwave radiation (see Fig 14) warms the upper water layers, leading to the reduction of mixed layer depth (as evident in Fig 12D) and decreasing temperature below it. The subsurface cooling below the warmed mixed layer reduces vertical mixing in the upper thermocline and redistributes nutrients apart from the heat and salt [51].

It is interesting to see that the chlorophyll concentration in the Strait of Malacca is very prominent too. From Eq (8), the resonance frequency at the head of the Strait of Malacca (see Fig 1C, dashed line: T2) can be estimated to be around 11.3 hours. This value is roughly close to the periods of M_2_ (12.4 hours) and S_2_ (12 hours) due to the narrow channel (approximately 350 km width and 120 m deep) feature here. As such, very strong mixing is effected by semidiurnal tides, mostly by M_2_ and to a lesser extent by S_2_ tides. Further work on this aspect will be done in future.

## Summary and Conclusions

Results of the simulated tides in the Continental Shelf Area of the SSCS from a 3D, one-way nested regional ocean modelling system indicate that the modelled tides compare well with the observations at 19 tidal stations. Existence of high tidal elevation at the southern tip of the ECPM and East Malaysia reflect the significant role of tides in these regions. Moreover, the M_2_ tidal current tends to flow towards ECPM due to the convergence of the counter clockwise tidal current from the coast of Vietnam and the clockwise tidal current from East Malaysia. Depth of the bathymetry can alter significantly the phase change. In general, the region between the southern end of the ECPM and coastal Sarawak is dominated by the diurnal tide with a counter clockwise phase rotation. However, along the coastal areas of East Malaysia, especially the coastal areas of Sarawak in places like Tanjung Datu, Kuching and Kuala Paloh are affected by the M_2_ tide with the high energy flux and strong tidal current rotating in a clockwise direction. The patterns of the co-tidal lines (lines of constant tidal phase) for the two main tidal constituents, namely M_2_ and K_1_ are the result of their differing amplitudes, leading to different contributions of tidal energies for each tidal constituent in the SSCS. The largest tidal energy flux in the study area is related mostly to the diurnal tides. There is a good correlation between the areas of high tidal energy flux and strong tidal current, causing the areas to be well mixed by the tidal mixing fronts. The areas affected significantly by the tidal mixing fronts are the coastal areas of Sarawak as exemplified by high chlorophyll concentrations. Nonetheless, more work needs to be done by considering the use of more refined model and bathymetry resolutions as well as more robust harmonic analysis with favourable hydrographic conditions.

## References

[1] Wyrtki K. Physical oceanography of the Southeast Asian water. In NAGA Report Vol. 2, Scientific Result of Marine Investigation of the South China Sea and Gulf of Thailand 1959–1961, Scripps Institution of Oceanography, La Jolla, California; 1961. pp.195.

[2] Shaw PT, Chao SY. Surface circulation in the South China Sea. Deep-Sea Res I. 1994; 40 (11/12): 1663–1683.

[3] Chao SY, Shaw PT, Wu SY. Deep water ventilation in the South China Sea. Deep-Sea Res I. 1996; 43 (4): 445–466.

[4] Chu PC, Edmons NL, Fan CW. Dynamical mechanisms for the South China Sea seasonal circulation and thermohaline variability. J. Phys. Oceanogr. 1999; 29: 2971–2989.

[5] Hu JY, Kawamura H, Hong H, Qi YQ. A review on the currents in the South China Sea: seasonal circulation, South China Sea warm current and Kuroshio intrusion. J. Oceanography. 2000; 56: 607–624.

[6] Daryabor F, Samah AA, Ooi SH, Chenoli SN. An estimate of the Sunda Shelf and the Strait of Malacca transports: a numerical study. Ocean Sci. Discuss. 2015a; 12(1): 275–313.

[7] Daryabor F, Samah AA, Ooi SH. Dynamical Structure of the Sea off the East Coast of Peninsular Malaysia. Ocean Dynam. 2015b; 65(1): 93–106.

[8] Daryabor F, Tangang F, Juneng L. Simulation of Southwest Monsoon Current Circulation and Temperature in the East Coast of Peninsular Malaysia. Sains Malays. 2014; 43(3): 389–398.

[9] Daryabor F, Tangang F, Juneng L. Hydrodynamic and Thermohaline Seasonal Structures of Peninsular Malaysia's eastern continental shelf sea. In EGU General Assembly Conference Abstracts. 2010; 12: 778.

[10] Akhir M, Daryabor F, Husain M, Tangang F, Qiao F. Evidence of Upwelling along Peninsular Malaysia during Southwest Monsoon. Open Journal of Marine Science. 2015; (5): 273–279. 10.4236/ojms.2015.53022

[11] Daryabor F, Ooi SH, Samah AA, Akbari A. Dynamics of the water circulations in the southern South China Sea and its seasonal transports. PLoS ONE. 2016; 11(7): e0158415 10.1371/journal.pone.0158415 27410682 PMC4943643

[12] Mohn C, Erofeeva S, Turnewitsch R, Christiansen B, White M. Tidal and residual currents over abrupt deep-sea topography based on shipboard ADCP data and tidal model solutions for three popular bathymetry grids. Ocean Dynam. 2013; 63(2): 195–208.

[13] Morozov EG. Semidiurnal internal wave global field. Deep-Sea Res I. 1995; 42: 135–148.

[14] Egbert GD, Ray RD. Estimates of M2 tidal energy dissipation from TOPEX Posiedon altimeter data. J. Geophys. Res. 2001; 106: 22475–22502.

[15] Niwa Y, Hibiya T. Three-dimensional numerical simulation of M2 internal tides in the East China Sea. J. Geophys. Res. 2004; 109 (C4): 1–14.

[16] Ye AL, Robinson IS. Tidal dynamics in the South China Sea. Geophys. J. Roy. Astron. Soc. 1983; 72: 691–707.

[17] Fang G, Kwok YK, Yu K, Zhu Y. Numerical simulation of principal tidal constituents in the South China Sea, Gulf of Tonkin and Gulf of Thailand. Cont. Shelf Res. 1999; 19: 845–869.

[18] Zu T, Gan J, Erofeeva SY. Numerical study of the tide and tidal dynamics in the South China Sea. Deep-Sea Res I. 2008; 55: 137–154.

[19] Green JAM, David TW. Non-assimilated tidal modeling of the South China Sea. Deep-Sea Res I. 2013; 78: 42–48.

[20] Shchepetkin A, McWilliams JC. The regional oceanic modeling system (ROMS): a split-explicit, free-surface, topography-following-coordinate ocean model. Ocean Modell. 2005; 9: 347–404.

[21] Smith WHF, Sandwell DT. Global seafloor topography from satellite altimetry and ship depth soundings. Science. 1997; 277: 1957–1962.

[22] Large WG, McWilliams JC, Doney SC. Oceanic vertical mixing: a review and a model with nonlocal boundary layer parameterization. Rev. Geophys. 1994; 32: 363–403.

[23] Marchesiello P, McWilliams JC, Shchepetkin A. Open boundary condition for long-term integration of regional oceanic models. Ocean Modell. 2001; 3: 1–21.

[24] Debreu L, Vouland C, Blayo E. AGRIF: Adaptive grid refinement in Fortran. Comput Geosci. 2008; 34(1): 8–13.

[25] Flather RA. A tidal model of the north-west European continental shelf. Memoires de la Societe Royale des Sciences de Liege. 1976; 6: 141–164.

[26] Chapman DC. Numerical treatment of cross-shelf open boundaries in a barotropic coastal model. J. Phys. Oceanogr. 1985; 15: 1060–1075.

[27] Orlanski I. A simple boundary condition for unbounded hyperbolic flows. J. Comput. Phys. 1976; 21: 252–269.

[28] Foreman MGG, Henry RF, Walters RA, Ballantyne VA. A finite element model for tides and resonance along the north coast of British Columbia. J. Geophys. Res. 1993; 98: 2509–2531.

[29] Egbert GD, Erofeeva SY. Efficient Inverse Modeling of Barotropic Ocean Tides. J. Atmos. Oceanic Tech. 2002; 19: 183–204.

[30] Da Silva AM, Young CC, Levitus S. Atlas of Surface Marine Data 1994, Vol. 1, Algorithms and Procedures, technical report. Technical report, National Oceanographic and Atmospheric Administration, Silver Spring, Md. 1994.

[31] Antonov JI, Locarnini RA, Boyer TP, Mishonov AV, Garcia HE. World Ocean Atlas 2005, Volume 2: Salinity. S. Levitus, Ed. NOAA Atlas NESDIS 62, U.S. Government Printing Office, Washington, D.C; 2006, pp. 182.

[32] Locarnini RA, Mishonov AV, Antonov JI, Boyer TP, Garcia HE. World Ocean Atlas 2005, Volume 1: Temperature. S. Levitus, Ed. NOAA Atlas NESDIS 61, U.S. Government Printing Office, Washington, DC; 2006. pp. 182.

[33] Pawlowicz R, Beardsley R, Lentz S. Classical tidal harmonic analysis including error estimates in MATLAB using T_TIDE. Comput. Geosci. 2002; 28: 929–937.

[34] Berens P. CircStat: A MATLAB Toolbox for Circular Statistics. J. Stat. Softw. 2009; 31: 1–21.

[35] Janekovic I, Powell B. Analysis of imposing tidal dynamics to nested numerical models. Cont. Shelf Res. 2012; 34: 30–40.

[36] Moum JN, Caldwell DR, Nash JD, Gunderson GD. Observations of boundary mixing over the continental slope. J. Phys. Oceanogr. 2002; 32: 2113–2130.

[37] Yanagi T, Takao T. Clockwise phase propagation of semidiurnal tides in the Gulf of Thailand. J. Oceanography. 1998; 54(2): 143–150.

[38] Jan S, Chern CS, Wang J. Transition of tidal waves from the East to South China Seas over the Taiwan Strait: Influence of the abrupt step in the topography. J. Oceanography. 2002; 58(6): 837–850.

[39] An HS. A Numerical Experiment of the M2 Tide in the Yellow Sea. Journal of the Oceanographical Society of Japan. 1977; 33: 103–110.

[40] Davies AM, Kwong SCM, Flather RA. Formulation of a variable-function three-dimensional model, with application to the M2 and M4 tide on the North-West European Continental Shelf. Cont. Shelf Res. 1997; 17: 165–204.

[41] Guo X, Yanagi T. Three-Dimensional Structure of Tidal Current in the East China Sea and the Yellow Sea. J. Oceanography. 1998; 54: 651–668.

[42] Mao Q, Qi Y, Shi P, Zhan H, Gan Z. Is there any amphidromic point of S2 constituent around the Natuna Islands in the southern South China Sea?. Chinese Sci. Bull. 2006; 51(2): 26–30.

[43] Bowden KF. Physical Oceanography of Coastal Waters. Ellis Horwood Ltd., Chichester, UK; 1983. 302p.

[44] Ding Y, Bao X, Yu H, Kuang L. A numerical study of the barotropic tides and tidal energy distribution in the Indonesian seas with the assimilated finite volume coastal ocean model. Ocean Dynam. 2012; 62(4): 515–532.

[45] Ray RD, Egbert GD, Erofeeva SY. A brief overview of tides in the Indonesian Seas. J. Oceanography. 2005; 18(4): 74–79.

[46] Simpson JH, Hunter JR. Fronts in the Irish Sea. Nature. 1974; 250: 404–406.

[47] Yanagi T, Sachoemar IS, Takao T, Fujiwara S. Seasonal Variation of Stratification in the Gulf of Thailand. J. Oceanography. 2001; 57: 461–470.

[48] Hu JY, Kawamura H, Tang DL. Tidal front around the Hainan Island, northwest of the South China Sea. J. Geophys. Res. 2003; 108(C11): 1978–2012.

[49] Yao Z, He R, Bao X, Wu D. M2 tidal dynamics in Bohai and Yellow Seas: a hybrid data assimilative modeling study. Ocean Dynam. 2012; 62(5): 753–769.

[50] Lorbacher K, Dommenget D, Niiler PP, Kohl A. Ocean mixed layer depth: A subsurface proxy of ocean-atmosphere variability. J Geophys Res-Ocean. 2006; 111(C7): 1978–2012. 10.1029/2003JC002157

[51] Nakamoto S, Prasanna KS, Oberhuber JM, Muneyama K, Frouin R. Chlorophyll Modulation of Sea Surface Temperature in the Arabian Sea in a Mixed-Layer Isopycnal General Circulation Model. J. Geophys. Res. Lett. 2000; 27(6): 747–750.

